# Altered proteostasis in aging and heat shock response in *C. elegans* revealed by analysis of the global and de novo synthesized proteome

**DOI:** 10.1007/s00018-014-1558-7

**Published:** 2014-01-24

**Authors:** Vanessa Liang, Milena Ullrich, Hong Lam, Yee Lian Chew, Samuel Banister, Xiaomin Song, Thiri Zaw, Michael Kassiou, Jürgen Götz, Hannah R. Nicholas

**Affiliations:** 1 Brain and Mind Research Institute, University of Sydney, Camperdown, 2050 Australia; 2 Institute for Integrative Neuroanatomy, Charité, Universitätsmedizin Berlin, Berlin, Germany; 3 School of Molecular Bioscience, University of Sydney, Building G08, Sydney, 2006 Australia; 4 Drug Discovery Research Laboratory, Brain and Mind Research Institute, University of Sydney, Camperdown, 2050 Australia; 5 Australian Proteome Analysis Facility, Macquarie University, Sydney, 2109 Australia; 6 Clem Jones Centre for Ageing Dementia Research (CJCADR), Queensland Brain Institute (QBI), The University of Queensland, Brisbane, 4072 Australia

**Keywords:** Click chemistry, Heat shock proteins, iTRAQ quantitative mass spectrometry, Ribosomal proteins, Aging, *C. elegans*

## Abstract

Protein misfolding and aggregation as a consequence of impaired protein homeostasis (proteostasis) not only characterizes numerous age-related diseases but also the aging process itself. Functionally related to the aging process are, among others, ribosomal proteins, suggesting an intimate link between proteostasis and aging. We determined by iTRAQ quantitative proteomic analysis in *C. elegans* how the proteome changes with age and in response to heat shock. Levels of ribosomal proteins and mitochondrial chaperones were decreased in aged animals, supporting the notion that proteostasis is altered during aging. Mitochondrial enzymes of the tricarboxylic acid cycle and the electron transport chain were also reduced, consistent with an age-associated energy impairment. Moreover, we observed an age-associated decline in the heat shock response. In order to determine how protein synthesis is altered in aging and in response to heat shock, we complemented our global analysis by determining the de novo proteome. For that, we established a novel method that enables both the visualization and identification of de novo synthesized proteins, by incorporating the non-canonical methionine analogue, azidohomoalanine (AHA), into the nascent polypeptides, followed by reacting the azide group of AHA by ‘click chemistry’ with an alkyne-labeled tag. Our analysis of AHA-tagged peptides demonstrated that the decreased abundance of, for example, ribosomal proteins in aged animals is not solely due to degradation but also reflects a relative decrease in their synthesis. Interestingly, although the net rate of protein synthesis is reduced in aged animals, our analyses indicate that the synthesis of certain proteins such as the vitellogenins increases with age.

**Electronic supplementary material:**

The online version of this article (doi:10.1007/s00018-014-1558-7) contains supplementary material, which is available to authorized users.

## Introduction

The cellular protein homeostasis (proteostasis) machinery regulates protein translation, folding, trafficking, and degradation in a highly coordinated manner, thereby ensuring the maintenance of a functional proteome [1]. However, as cells age, there is both an increased misfolding of proteins and an increased accumulation of misfolded proteins, the latter in part because their clearance is impaired. Protein aggregation is particularly relevant in age-associated neurodegenerative conditions such as Alzheimer’s disease (AD) [2], for which a failure of proteostasis with aging has been suggested as an initiating factor [3]. Although it is not fully understood what causes the age-associated decline in proteostasis, an intimate link between proteostasis in general and aging is evident, as supported by studies in the nematode *Caenorhabditis elegans* (reviewed in [4]).

Heat shock proteins (HSPs) are critical contributors to proteostasis. The HSPs are chaperones with an essential role in the proper folding of newly synthesized proteins and in preventing their premature interaction with other proteins. Under conditions of stress (with heat shock being a widely used experimental paradigm) heat shock proteins are rapidly upregulated and bind to partially unfolded proteins, thereby preventing misfolding and aggregation. The heat shock pathway is also important for aging, as demonstrated by the fact that reducing the activity of the transcription factor HSF-1, which regulates the heat shock response, accelerates tissue aging and shortens life-span in *C. elegans* [5]. Conversely, lifespan extension, i.e., deceleration of aging, can be achieved in *C. elegans* by increasing the expression of a small HSP, HSP-16 [6]. The dye Thioflavin T that stains protein aggregates in AD brain, promotes protein homeostasis in vivo and increases nematode longevity, and these beneficial effects depend, among others, on HSF-1 [7].

In addition to the heat shock pathway, the protein translation machinery has been implicated as a regulator of aging. In *C. elegans*, RNAi-mediated depletion of several translation initiation factors, ribosomal proteins and regulators of translation extends lifespan significantly [8–10]. Age-associated changes in proteostasis are further reflected in the observation that protein aggregation increases with age in *C. elegans*, and seems to be an inherent property of a vast array of proteins [11, 12].

Mitochondria are also important regulators of aging, as indicated, for example, by observations that reduced function of components of the electron transport chain (ETC) extends lifespan in *C. elegans* and other organisms [13–16]. These mitochondrial perturbations result in the induction of the mitochondrial unfolded protein response (UPR), characterized by increased expression of the mitochondrial chaperones HSP-6 (mt Hsp70) and HSP-60 (mt chaperonin) [17–22]. Similarly, the mitochondrial UPR is induced and nematode lifespan is increased by reduced function of mitochondrial ribosomal proteins [23]. These findings highlight the importance of mitochondrial proteostasis in longevity (reviewed in [24]).

Because of the intimate link between aging and the heat stress response, we set out to apply quantitative proteomics to analyze the aging proteome and the proteomic response to heat stress, and to uncover how this response changes with age. In addition to this global analysis, we specifically determined the pool of de novo synthesized proteins. To this end, we developed novel protocols for labeling and visualizing newly synthesized proteins in *C. elegans*. These protocols are based on two previously established in vitro methods, bio-orthogonal non-canonical amino acid tagging (BONCAT) for detection by Western blotting [25, 26], and fluorescent non-canonical amino acid tagging (FUNCAT) for detection by fluorescence microscopy [27]. Both methods depend on the incorporation of a methionine derivative, azidohomoalanine (AHA). The frequency of methionine in *C. elegans* proteins is in the order of only 2.5 % [28], which presents a significant advantage because toxicity due to the incorporation of AHA is minimized. Likewise, a low charging rate further reduces the potential toxicity of AHA. Importantly, AHA’s azide group can be selectively reacted with either an alkyne-labeled fluorescent dye or biotin, thereby enabling visualization of labeled proteins by fluorescence microscopy or Western blotting, respectively. These azide-alkyne cycloaddition reactions are examples of ‘click chemistry’, a term coined by Sharpless and colleagues [29] to describe high-yielding, modular reactions that generate heteroatom links (C–X–C) and only inoffensive byproducts. As an add-on to our global proteome analysis, we identified AHA-tagged proteins using iTRAQ (Isobaric Tags for Relative and Absolute Quantitation) quantitative mass spectrometry, a method we have previously used to determine global proteomic changes quantitatively [30].

Our investigation reveals significant age-associated changes in the nematode proteome and in the capacity of nematodes to respond to heat stress. In addition to impaired protein clearance in aged animals, reduced rates of protein synthesis have previously been shown [31, 32]. Our unbiased global proteomics approach supports this observation, demonstrating most notably a marked age-dependent decrease in the abundance of numerous ribosomal proteins, together with reduced levels of several mitochondrial chaperone proteins. We extended our observations by additionally examining the AHA-tagged pool of de novo synthesized proteins and found specific changes to protein synthesis in aged animals.

## Experimental procedures

### Strain information

The *C. elegans* wild-type strain N2 variety Bristol and the *E. coli* strains OP50 and HB101 were obtained from the Caenorhabditis Genetics Center (CGC, University of Minnesota, Twin Cities, MN, USA). Nematodes at the first larval (L1) stage were transferred to nematode growth medium agar plates. After incubation for 24 h at 25 °C, they were washed off the plates with S-medium [33] and transferred into 50 ml of liquid culture medium (S-medium supplemented with 300 U/ml of nystatin (Sigma-Aldrich, St. Louis, MO, USA) and 50 μg/ml of streptomycin (Sigma), using concentrated HB101 bacteria for feeding) and then grown at 20 °C. Samples from the culture were monitored daily using a dissecting microscope to observe the developmental stage and numbers of worms, until ≈220,000 were obtained (at approximately the 8th day of culture). Gravid adult worms were collected in 50-ml tubes and centrifuged with a low brake setting at 480 × *g* for 3 min at 15 °C. The supernatant was discarded and approximately 1 ml of compact worms was transferred to 15-ml tubes. The nematodes were then synchronized using 7.5 % sodium hypochlorite, 1.6 M NaOH. The worms were vortexed in this bleaching solution for 2 min and the tube was then topped up with 10 ml of milli-Q water. After three washing steps, the nematodes were transferred into new flasks and hatched overnight (o/n) in liquid culture medium without HB101 to arrest at the L1 stage. On the following day, concentrated HB101 was added to the culture to reinitiate development. To prevent growth of progeny, 25 μM fluorodeoxyuridine (FUdR) (Sigma) was added as soon as the population had reached the fourth larval (L4) stage. Every second day, the nematodes were washed several times by sedimentation to remove any progeny, followed by resuspension in fresh medium.

### Heat shock, AHA labeling, and sampling

At the three stages young adult (YA, L4 + 12 h), Day 5 (D5, L4 + 6 days) and Day 10 (D10, L4 + 11 days), animals were washed in FUdR-containing M9 buffer [33], then cultures of up to 20,000 animals were set up, containing 2 mM AHA (4-azido-l-homoalanine; ABCR Karlsruhe, Germany) in S-medium together with OP50. For fluorescence analysis, 0.5 or 2 mM AHA was used. Heat shock (HS) was administered at 34 °C for 2 h with constant agitation, whereas controls were incubated at 20 °C. Both groups were recovered at 20 °C for 4 h. For analysis of AHA incorporation by fluorescence microscopy and Western blotting, control animals were incubated in S-medium with OP50 without AHA. After treatment and recovery, the cultures were transferred to 1.5-ml DNA-lo-binding tubes (Eppendorf, Sydney, Australia) and nematodes allowed to sediment at room temperature (RT) for 10–15 min. The supernatant was removed and the nematodes washed with S-medium. The nematodes were then incubated at RT for 30 min to allow digestion of residual bacteria in the gastrointestinal tract. After discarding the supernatant, distinct procedures were used to prepare samples for either fluorescence imaging, Western blotting, or proteomics.

### ‘Click chemistry’ for fluorescence microscopy

Nematode samples were prepared for fluorescence microscopy after AHA labeling using peroxide tube fixation as described [34]. Specifically, the worms in the DNA-lo-binding tubes were washed three times briefly in M9 buffer by sedimentation. All but 500 μl of the supernatant was removed after the final washing step. Then, 500 μl of ice-cold 2× MRWB (Modified Ruvkun’s Witches Brew): 160 mM KCl, 40 mM NaCl, 20 mM EDTA, 10 mM spermidine hydrochloride, 30 mM NaPIPES, 50 % methanol, 4 % formaldehyde in milli-Q water was added, followed by a brief vortexing step. The worms were frozen in liquid nitrogen and then defrosted. Following two additional freeze–thaw cycles, the samples were fixed o/n at 4 °C. Permeabilization was performed as described [35] with the exception that samples were finally washed in PBS with 0.5 % Triton X-100 for 15 min. More specifically, samples were spun at 10,000 rpm for 1 min to remove the fixative, followed by three washes in Tris-Triton buffer (0.1 M TRIZMA base minimum 99.9 %, pH 7.4, 1 % Triton X-100, 1 mM EDTA in milli-Q water) and one wash in 1× borate buffer (2.5 % 40× borate stock buffer, 0.01 % Triton X-100 in milli-Q water, pH adjusted to 9.5 with NaOH; 40× borate stock buffer: 1 M H_3_BO_3_, 0.5 M NaOH in milli-Q water, titrated to pH ≥ 9.5). The worms were then incubated in 1 ml of 1 % 2-mercaptoethanol in borate buffer for 2 h at 37 °C with vigorous shaking in a Thermomixer at a 1,400 rpm setting. After spinning at 3,000 rpm for 2 min, the supernatant was removed and the worms incubated in 1 ml 10 mM DTT (borate buffer) for 15 min at RT with gentle agitation, followed by one wash in 1× borate buffer. Then, the worms were first incubated in 0.3 % hydrogen peroxide (borate buffer) for 15 min at RT with gentle agitation, followed by one wash in 1× borate buffer, and then in 1× PBS with 0.5 % Triton X-100 for 15 min at RT.

A ‘click chemistry’ solution was prepared with final concentrations of 200 μM triazole ligand, 5 μM (fluorescent) Chromeo™-546-alkyne (BaseClick, Tutzing, Germany), 400 μM TCEP (Sigma), and 200 μM CuSO_4_ in PBS. After each addition, the ‘click chemistry’ reaction mix was vortexed vigorously. Negative controls were only reacted with 5 μM Chromeo™-546-alkyne in PBS (that is, without the triazole ligand, TCEP and CuSO_4_). Then, 250 μl of the ‘click chemistry’ solution was aliquoted into fresh 1.5-ml Eppendorf tubes to which 10 μl of fixed worms were added. Worms were incubated o/n at RT on a rotisserie rotator. Then, the samples were washed 4 times for 30 min in PBDTT (PBS with 1 % DMSO, 0.1 % Tween 20, 0.5 % Triton X-100) containing 0.5 mM EDTA, followed by two 1-h washes in PBDTT. Samples were then mounted onto slides using Fluoromount G (SouthernBiotech, Birmingham, AL, USA). A Zeiss LSM710 confocal microscope was used to collect images. De novo synthesized proteins tagged with Chromeo™-546-alkyne were excited with 561 nm and light captured between 560 and 600 nm.

### ‘Click chemistry’ for Western blotting

After AHA labeling, worms were sedimented for 10–15 min at RT or for 3 min on ice, and the supernatant discarded. Nematode pellets were washed with chilled PBS-MC (PBS, 1 mM MgCl_2_, 0.1 mM CaCl_2_) then with chilled PBS-MC-PI [PBS-MC with complete EDTA-free protease inhibitor (Roche, Penzberg, Germany)], with the supernatant being removed following centrifugation at 2,000* × g* for 5 min at 4 °C after each wash. The nematode pellets were then frozen at −80 °C. The pellets were defrosted on ice and 200 μl lysis buffer (0.5 % (w/v) SDS, 1 % Triton X-100 with complete EDTA-free protease inhibitor (Roche) in PBS) was added. The samples were sonicated 10× with 1 s pulses for eight cycles with a 20–40 % amplitude. Complete lysis of the worms was confirmed under the microscope. Then, 1 μl benzonase (>500 U, Sigma) was added and samples boiled for 10 min at 96 °C. After chilling on ice, 800 μl PBS was added, adjusting the solution to 0.1 % (w/v) SDS and 0.2 % (w/v) Triton X-100. Samples were centrifuged at 13,000* × g* for 10 min at 4 °C to remove debris. A total of 500 μl of the supernatant was transferred to Amicon Ultra 0.5-ml centrifugal tubes (Merck Millipore, Kilsyth, Australia) for protein concentration. More specifically, the samples were centrifuged at 14,000* × g* for 30 min at RT. Then, the filter unit was placed upside down in a new Amicon microcentrifuge tube. Proteins were recovered by spinning at 1,000* × g* for 2 min at RT. This centrifugation step was repeated twice, using the same filter unit for the remaining 500 μl of the supernatant. Then, the total concentrated protein sample was transferred into new 1.5-ml tubes. Only then, a 10-mg/ml copper bromide suspension in molecular biology grade water was prepared by vigorously vortexing for 20 s. For ‘click chemistry’, the following reagents were added to the concentrated sample by vortexing in this order: 1 μl 200 mM triazole ligand (Sigma); 2 μl 25 mM biotin-alkyne tag (biotin–PEG3–propargylamide); 10 μl 10 mg/ml copper (I) bromide suspension (Sigma) in molecular-grade water (Sigma). The samples were vortexed thoroughly for 15–20 s after each reagent was added. After incubation on a rotisserie rotator o/n at 4 °C samples subjected to ‘click chemistry’ appeared light-green whereas non-reacted controls remained colorless. The supernatant was collected after centrifuging at 2,000* × g* for 5 min at 4 °C. 30 μg protein was separated on a 10 % Tris–glycine gel, followed by transfer to a nitrocellulose membrane. To detect de novo synthesized proteins tagged with biotin, mouse anti-biotin-AP (Sigma) was used o/n at 4 °C, followed by incubation with goat anti-mouse-HRP (Santa Cruz, Texas, USA) antibody and reaction with HRP-substrate. Rabbit anti-actin (Sigma) was used as loading control.

### iTRAQ quantitative mass spectroscopy and data analysis

For full experimental details of proteomics analysis, see Supplementary methods. Briefly, nematode lysates were extracted and labeled with four iTRAQ 4-plex peptide labeling reagents (using isobaric tags 114, 115, 116, and 117). Four experimental runs were conducted, with aliquots of three pooled heat shocked young adult (YA) samples used in all runs as an internal control. In detail, in the first run, samples consisted of heat shocked and non-heat shocked YA and D5 adults. In the second experiment, samples consisted of two heat shocked YA samples (one being the internal control) as well as heat shocked and non-heat shocked D10 adults. The third experiment ran the control sample together with non-heat shocked YAs and heat shocked and non-heat shocked D5 adults. The fourth experiment consisted of the control sample as well as two samples of heat shocked D10 adults and one sample of non-heat shocked D10 adults. The samples were pooled and fractionated prior to analysis via nanoLC-ESI–MS/MS. Data were analyzed using ProteinPilot V4.2 (AB Sciex).

## Results

### Proteomic analysis of aging and the heat shock response in *C.* *elegans*

We set out to examine the relationship between proteostasis and aging in *C.* *elegans* using quantitative proteomics. In addition to profiling the aging proteome by investigating three age groups, we examined the heat shock response during aging, since the heat shock pathway is not only important for aging, but because heat shock is also a well-established experimental paradigm in organisms ranging in complexity from yeast to mammals [36, 37]. Alongside a global proteomic analysis, we were particularly interested in the pool of newly synthesized proteins, and how this de novo proteome changes with age and under conditions of stress.

**Fig. 1:**
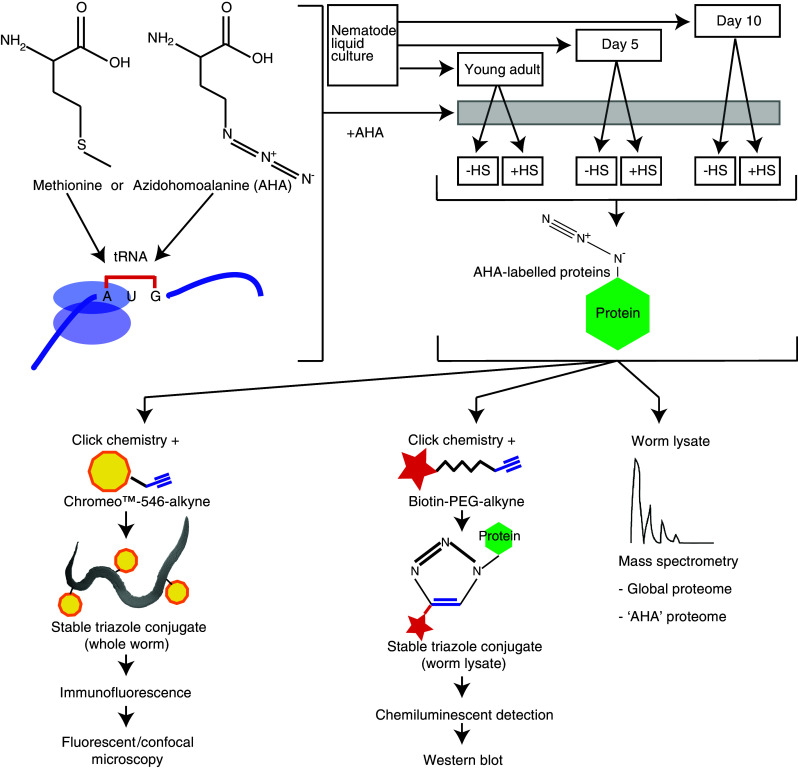
AHA labeling of de novo synthesized proteins in *C. elegans*. Schematic showing incorporation of AHA during aging and heat shock along with subsequent analysis of AHA-tagged proteins

To profile the aging proteome and enable a comparison between early adulthood and aged animals, three representative time points were selected: day 1 of adulthood (young adult/YA), day 5 (D5) and day 10 (D10). For each time point, two populations of nematodes were examined; one grown at 20 °C and another subjected to heat shock for 2 h at 34 °C followed by 4-h recovery at 20 °C (Fig. 1).

### De novo synthesized proteins can be visualized by bio-orthogonal labeling in vivo

**Fig. 2:**
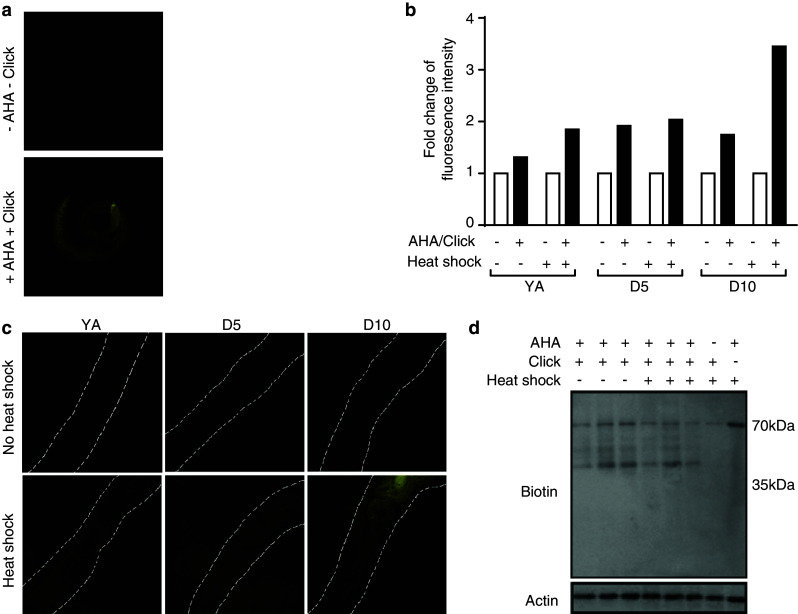
AHA-labeled proteins in *C. elegans* can be visualized by immunofluorescence analysis or immunoblot after ‘click chemistry’ using Chromeo-546-alkyne or biotin-alkyne tags, respectively. Animals were incubated with 0.5 or 2 mM (for immunofluorescence) or 2 mM AHA (for immunoblot) for 6 h before ‘click chemistry’ was performed at either young adult (YA), day 5 (D5) or day 10 (D10) of adulthood. All samples, including controls, were incubated with the Chromeo™-546-alkyne or biotin-alkyne tags. **a** Representative fluorescence images showing 2 mM AHA-labeled and unlabeled nematodes. The *top panel* “−AHA −Click” refers to sample where no AHA was added and ‘click chemistry’ was not performed. The *bottom panel* “+AHA +Click” refers to a where ‘click chemistry’ was performed on a sample where AHA had been added. **b** Fold changes of fluorescence intensity (in arbitrary units, measured using ImageJ software) between AHA-labeled and non-labeled nematodes at YA, D5, or D10 of adulthood, with or without heat shock treatment. In this experiment, labeling was conducted with 0.5 mM AHA and ‘click chemistry’ was performed only when AHA was added. **c** Representative fluorescence images at 546 nm of 0.5 mM AHA-labeled (+‘click chemistry’) non-heat shocked and heat shocked nematodes shown at YA, D5, or D10 of adulthood. **d** Immunoblot detection of biotin-labeled AHA-tagged proteins in YA animals in non-heat shocked and heat shocked samples. Actin was used as loading control. The *rightmost two lanes* are control samples indicating the absence of bands (other than a non-specific signal at 70 kDa) when no AHA is added before ‘click chemistry’, or when AHA is added but ‘click chemistry’ is not performed

To enable the identification of de novo synthesized proteins, we established protocols for bio-orthogonal tagging of proteins in *C. elegans* using the methionine homologue AHA. Metabolic labeling of proteins with AHA has previously been reported in cultured mammalian cells [25] and in larval zebrafish [38], but not *C. elegans*. We therefore first delineated suitable conditions for AHA incorporation into proteins in adult nematodes, growing them for 6 h at a range of concentrations (0–2 mM). After fixation and permeabilization, fluorescent Chromeo-546-alkyne was applied and reacted with AHA using ‘click chemistry’, and subsequently detected by confocal microscopy. Compared with untreated controls, where the alkyne tag was added, AHA was not added, and no ‘click chemistry’ was performed, increased fluorescence was detected in all tissues of nematodes grown in the presence of AHA, indicating successful incorporation (Fig. 2a). Thus, such an approach enables the visualization of de novo protein synthesis at a cellular level in response to any insult or stimulus. Toxicity as determined by impaired thrashing was only found at higher AHA concentrations and for prolonged incubation times (Online resource 1a and data not shown). To specifically assess whether AHA exposure might initiate the mitochondrial UPR, a *hsp*-*6::gfp* reporter [21] was monitored. No change in expression of this reporter following a 6-h incubation in 2 mM AHA was observed (Online resource 1b).

We next tested whether AHA can be incorporated at the relevant life stages and under conditions of heat stress. We cultured YA, D5 and D10 nematodes with and without 0.5 mM AHA and subjected them either to control conditions or heat shock. Following reaction with Chromeo-546-alkyne, increased fluorescence was detected in all samples grown with AHA (Fig. 2b, c).

As a complementary approach to visualize AHA incorporation, Western blotting was used. We established suitable conditions by obtaining protein lysates from nematodes that had been grown for 6 h in 0–8 mM AHA. ‘Click chemistry’ was used to react the incorporated AHA with biotin that was, in contrast with the BONCAT protocol [25], directly coupled to an alkyne-reactive group. The samples were analyzed by Western blotting, using an anti-biotin antibody to visualize AHA-tagged proteins. This established that AHA incorporation is detectable at all tested concentrations (data not shown). We next examined incorporation into YA, D5 and D10 nematodes under both control and heat shock conditions. For all conditions tested, AHA was incorporated into proteins over a wide range of molecular weights (Fig. 2d). A 70-kDa band was observed in all samples even in the absence of AHA labeling and ‘click chemistry’, indicating cross-reactivity with an unlabeled nematode protein. While incubation time- and AHA concentration-dependent changes in relative AHA incorporation can be visualized using Western blotting (data not shown), this method is not sufficiently sensitive to detect age-dependent or heat shock-dependent changes in AHA incorporation (Fig. 2d and data not shown). We therefore proceeded to identifying de novo synthesized proteins by direct detection of the incorporated AHA using proteomics.

### iTRAQ analysis identifies 3,387 unique proteins

To prepare samples for quantitative proteomic analysis, nematodes were cultured in bulk until the relevant time points, and then split into ‘control’ and ‘heat shock’ samples. Two mM of AHA was added before heat shock commenced. Following a 2-h heat shock and 4-h recovery (6 h at 20 °C for the control), nematode samples were harvested. Fifteen samples including a minimum of two biological replicates for each of the six tested conditions were analyzed in four iTRAQ 4-plex experiments. To enable comparisons, one sample (‘young adult, heat shocked’, YAHS) served as an internal reference and was included in all four mixtures. iTRAQ-labeled samples were fractionated and subjected to nanoLC-ESI–MS/MS mass spectrometry. The data were analyzed using ProteinPilot V4.2 software with reference to the UniProt database collection from both *C. elegans* and *E. coli*.

To determine the global proteome, AHA modification was not included in the database search parameters. A total of 3,387 unique proteins from 20,182 unique peptides were identified, with an estimated protein identification false discovery rate of 0.15 %. Less than 0.2 % of all identified proteins were derived from the nematodes’ bacterial food source.

For the nematode proteins, the relative abundance for each condition was determined as the geometric mean of the iTRAQ ratios from replicate samples and the *p* value of this combined ratio was calculated according to Stouffer’s *z* test method. Proteins for which the combined ratio was <0.83 or >1.2, with *p* values <0.05, were considered to be either significantly less or more abundant. The changes observed were in the order of up to 25-fold. The thresholds were set with reference to the literature suggesting that iTRAQ in fact underestimates fold changes such that, for example, an iTRAQ ratio of 1.2 may reflect an actual twofold difference [39].

### The global proteome of aged animals is characterized by a relative abundance of vitellogenins and diminished levels of ribosomal, mitochondrial, and myosin-related proteins

**Fig. 3:**
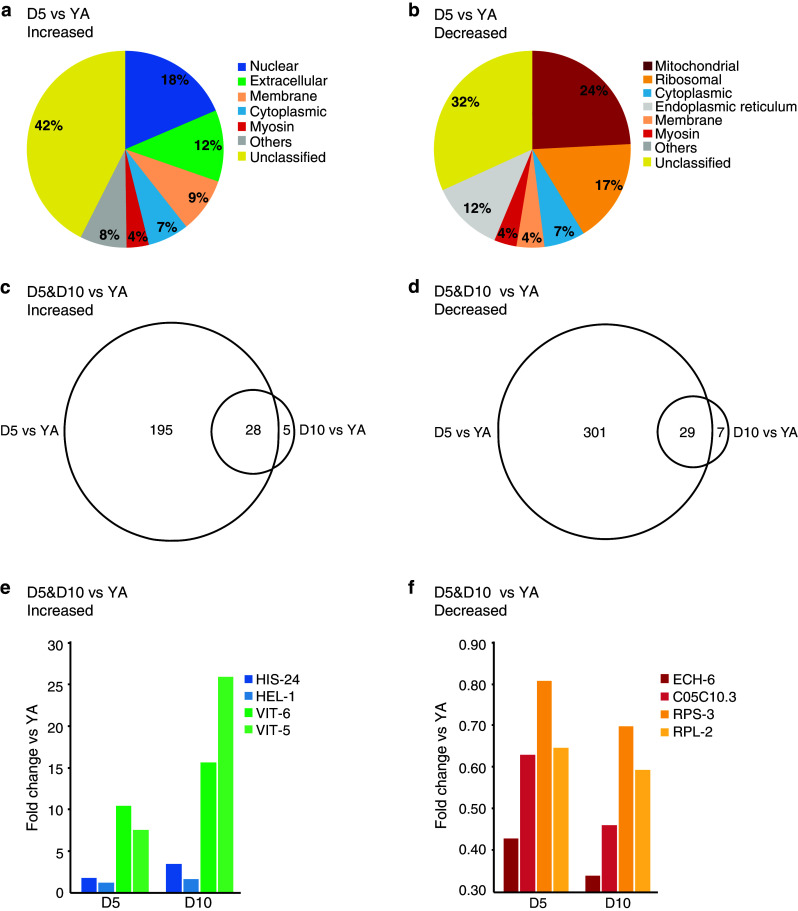
The proteomic profile of *C. elegans* changes with age. **a** Gene Ontology (cellular component) profile of proteins increased at D5 compared with YA. The extracellular proteins include vitellogenins. Only the major categories are shown; categories with fewer proteins are cumulatively displayed as ‘Others’. Proteins without a Gene Ontology (cellular component) classification are shown as ‘Unclassified’. **b** Gene Ontology (cellular component) profile of proteins decreased at D5 compared with YA. **c** Venn diagram of proteins increased at D5 and D10 compared with YA. **d** Venn diagram of proteins decreased in abundance at D5 and D10 compared with young adults (YA). Selected proteins that are increased (**e**) or decreased (**f**) in abundance at D5 and D10 compared with YA

We first considered the aging proteome. When comparing D5 aged adults with YA, 221 proteins were increased in abundance (Online Resource 2a). When we used Wormbase to classify the Gene Ontologies of these 221 increased proteins at the level of subcellular structures and macromolecular complexes (i.e., cellular components) we found nuclear and extracellular as the two most represented categories, with 41 and 26 proteins, respectively (Fig. 3a; Table 1). Among the identified nuclear proteins were replication licensing factors (MCM-2–7), DNA topoisomerase TOP-2, nuclear lamin LMN-1 and several histone proteins including HIS-1, -4, -35, and -71, and the histone H1 variant, HIS-24. The latter is involved in the regulation of immune-related genes [40]. Increased were also several extracellular proteins that belong to the Transthyretin-Related family (TTR-2, -15, -16, -45, and -51). A major increased group of proteins within the extracellular category are the yolk proteins, vitellogenins, which are required for oocyte development. Importantly, levels of all six vitellogenins (VIT-1–6) were elevated in the aged animals.

**Table 1:** Proteins increased in abundance in day 5 animals compared with young adults

UniProt accession	Gene	Name	Fold change
Extracellular			
Vitellogenins			
P55155	vit-1	Vitellogenin-1	3.5
P05690	vit-2	Vitellogenin-2	11.1
Q9N4J2	vit-3	Vitellogenin-3	5.6
P18947	vit-4	Vitellogenin-4	4.7
P06125	vit-5	Vitellogenin-5	7.5
P18948	vit-6	Vitellogenin-6	10.4
Transthyretins			
P34500	ttr-2	Transthyretin-like protein 2	3.7
O17345	ttr-6	Transthyretin-like protein 6	2.9
Q22288	ttr-15	Transthyretin-like protein 15	3.2
Q2EEM8	ttr-45	Transthyretin-like protein 45	3.5
O62289	ttr-51	Transthyretin-like protein 51	5.0
Chitin metabolic processes			
Q11174	cht-1	Probable endochitinase 1	5.7
Q18143	cht-3	Probable endochitinase 3	3.7
P41996	cpg-2	Chondroitin proteoglycan-2	3.0
Q18529	C39D10.7	Orthologous to human mucin-2	3.7
Others			
Q21265	cri-2	Conserved regulator of innate immunity, orthologous to human metalloproteinase inhibitor	2.5
Q20224	lbp-2	Fatty acid-binding protein homolog 2	2.1
G5EFP4	sym-1	Synthetic lethal with mec	2.3
Q18594	C44B7.5	Uncharacterized protein C44B7.5	4.8
O62053	C08F11.11	Uncharacterized protein UPF0375	3.6
Nuclear			
DNA replication and cell cycle			
P34556	cdk-1	Cyclin-dependent kinase 1	2.6
Q9XXI9	mcm-2	Mini-chromosome maintenance protein 2	2.9
Q9XVR7	mcm-3	Mini-chromosome maintenance protein 3	2.6
Q21902	mcm-5	Mini-chromosome maintenance protein 5	2.5
P34647	mcm-6	Mini-chromosome maintenance protein 6	2.7
O16297	mcm-7	Mini-chromosome maintenance protein 7	2.9
O02115	pcn-1	Proliferating cell nuclear antigen	3.5
P53016	rfc-4	Replication factor C subunit 4	3.7
Q19537	rpa-1	Replication protein A homolog	2.0
Q95Y97	rpa-2	Replication protein A homolog	2.0
Q19555	scc-3	Cohesin complex subunit	1.9
Q23670	top-2	DNA topoisomerase	2.0
Histones			
P62784	his-1	Histone H4	1.8
Q27894	his-4	Histone H2B2	3.1
P10771	his-24	Histone H1.1	1.8
Q10453	his-71	Histone H3.3 type 1	2.7
Transport			
P91276	ima-2	Importin subunit alpha-2	1.8
O17915	ran-1	GTP-binding nuclear protein	1.8
Others			
B6VQ74	F08B12.4	Uncharacterized protein F08B12.4, isoform a	2.1
G5EF53	swsn-4	SWI/SNF nucleosome remodeling complex component	1.9

Proteins showing the greatest fold changes from among the cellular component categories “extracellular” and “nuclear” are listed

We next considered the group of proteins that is decreased in abundance with age. A total of 327 proteins were reduced at D5 compared with YA (Online Resource 2b). Among these decreased proteins, the two most represented cellular components are mitochondrial and ribosomal, with 79 and 56 proteins, respectively (Fig. 3b; Table 2). The decreased mitochondrial proteins include 20 components of the ETC and 14 enzymes of the tricarboxylic acid (TCA) cycle. Also decreased were several factors that play roles in mitochondrial proteostasis. These include Hsp70 (HSP-6), the co-chaperone grpE (C34C12.8), Tim44 (T09B4.9), Hsp60 (HSP-60), Hsp90 (R151.7a), clpX (D2030.2), two mitochondrial translation factors (GFM-1 and TSFM-1), a mitochondrial ribosomal protein (MRPL-12) and structural proteins (ATAD-3 and the prohibitins PHB-1 and PHB-2) (reviewed in [24]).

**Table 2:** Proteins decreased in abundance in day 5 animals compared with young adults

UniProt accession	Gene	Name	Fold change
Mitochondrial			
Electron transport chain			
Q19126	asb-2	ATP synthase B homolog	0.4
Q18803	asg-2	ATP synthase subunit G2	0.5
P46561	atp-2	ATP synthase subunit beta	0.6
O16517	atp-4	ATP synthase subunit	0.5
G5EDD1	ucr-2.1	Ubiquinol-cytochrome c oxidoreductase complex	0.4
Q22370	ucr-2.2	Ubiquinol-cytochrome c oxidoreductase complex	0.6
Chaperones			
P11141	hsp-6	Heat shock 70-kDa protein F, Hsp70 family	0.6
P50140	hsp-60	Chaperonin homolog, Hsp60 family	0.6
P90788	D2030.2	Orthologous to human ATP-dependent Clp protease ATP-binding subunit clpX-like, Hsp100 family	0.5
Fatty acid metabolic processes			
H2KZG6	acdh-1	Acyl CoA dehydrogenase	0.2
O18693	acs-2	Fatty acid CoA synthetase family	0.6
Q9BI69	alh-13	Aldehyde dehydrogenase	0.5
P34559	ech-6	Enoyl-CoA hydratase	0.4
Tricarboxylic acid (TCA) cycle-related			
P34575	cts-1	Citrate synthase	0.6
O44451	pdhb-1	Pyruvate dehydrogenase beta	0.6
Q09545	sdhb-1	Succinate dehydrogenase complex subunit B	0.5
Others			
P54688	bcat-1	Branched-chain-amino-acid aminotransferase	0.6
Q18885	icd-1	Inhibitor of cell death; orthologous to human beta-subunit of the nascent polypeptide-associated complex	0.5
Q21752	vdac-1	Voltage-dependent anion-selective channel homolog	0.6
O45011	W10C8.5	Orthologous to human isoform 1 of creatine kinase U-type, mitochondrial	0.5
Ribosomal			
Large subunit			
O02056	rpl-4	60S ribosomal protein L4	0.6
Q9XVE9	rpl-14	60S ribosomal protein L14	0.6
P34334	rpl-21	60S ribosomal protein L21	0.5
P52819	rpl-22	60S ribosomal protein L22	0.6
P48162	rpl-25.1	60S ribosomal protein L23a 1	0.4
Q9BKU5	Y37E3.8	60S ribosomal protein L27a	0.6
P49181	rpl-36	60S ribosomal protein L36	0.5
P48166	rpl-41	60S ribosomal protein L44	0.6
Small subunit			
P48156	rps-8	40S ribosomal protein S8	0.6
P51404	rps-13	40S ribosomal protein S13	0.5
O01692	rps-17	40S ribosomal protein S17	0.6
O18240	rps-18	40S ribosomal protein S18	0.5
O18650	rps-19	40S ribosomal protein S19	0.6
Q8WQA8	rps-20	40S ribosomal protein S20	0.6
Q1XFY9	rps-24	40S ribosomal protein S24	0.6
P52821	rps-25	40S ribosomal protein S25	0.5
P37165	ubl-1	Ubiquitin-like protein 1, 40S ribosomal protein S27a	0.6
Others			
O01504	C37A2.7	60S acidic ribosomal protein P2	0.5
O18180	mrpl-12	Mitochondrial Ribosomal Protein, Large	0.6
Q93572	rpa-0	60S acidic ribosomal protein P0	0.6

Proteins showing the greatest fold changes from among the cellular component categories “mitochondrial” and “ribosomal” are listed

The decreased ribosomal proteins at D5 compared with YA include 22 components of the small (40S) ribosomal subunit and 28 components of the large (60S) ribosomal subunit. This striking decrease in abundance of numerous ribosomal subunits in aged animals is consistent with the observation of a decrease in protein synthesis during aging [31]. Other decreased proteins that contribute to the regulation of protein synthesis include RACK-1, a scaffolding component of the 40S ribosomal subunit (reviewed in [41]) and C08H9.2, the nematode ortholog of vigilin, which associates with 80S ribosomes and is proposed to regulate the translocation of tRNAs from the nucleus to the cytoplasm for association with ribosomes [42]. Among the decreased proteins were also a translation initiation factor (eIF5A homologue, IFF-2), a translation elongation factor (EF-2 homologue, EEF-2) and a polyA binding protein (PAB-1).

Outside these two major categories of decreased proteins, 12 myosin-related proteins were also decreased in D5 aged animals, including myosin heavy chain isoforms (MYO-1, -2, -3, UNC-54), myosin light chain isoforms (MLC-1, MLC-3), troponin T (MUP-2), tropomyosin (LEV-11), and paramyosin (UNC-15).

Since 42 % (94/221) of proteins that were increased and 32 % (104/327) of proteins that were decreased in D5 aged animals did not have a Gene Ontology (cellular component) term listed in WormBase (identified as “Unclassified” in Fig. 3a, b), we extended our analysis by identifying the human orthologs of these proteins and classifying the proteins based on the cellular component Gene Ontologies of these orthologs (Online Resource 2a and 2b). Similar to the primary analysis described above, the most represented categories among the increased proteins were nuclear, cytoplasmic, extracellular and membrane, while cytoplasmic and mitochondrial were most represented among the decreased proteins (Online Resource 2c).

How does the proteome change when we analyze even older worms? We next compared D10 adults with YA. Here, relative protein abundance was calculated indirectly since these conditions were not assayed in the same iTRAQ 4-plex experiment. That is, the D10 versus YAHS and YA versus YAHS ratios were calculated directly and then the former were divided by the latter to yield the D10 versus YA ratio. A *p* value was calculated using a Student’s *t* test and *p* < 0.05 was considered significant. Using this method, 33 proteins were identified as increased in abundance at D10 compared with YA, while 36 proteins were decreased in abundance (Online Resource 3a–3c). Although the number of proteins showing changes in abundance at D10 is much smaller than those identified at D5, this most likely reflects the more stringent statistics applied to these indirectly calculated data, rather than suggesting that the proteome of D10 adults is more similar to YA than that of D5 adults is. Importantly, Wormbase analysis of the Gene Ontologies of the proteins that were increased and decreased at D10 compared with YA revealed enrichment of the same cellular components as had been observed in the D5 proteome. That is, nuclear and extracellular proteins are most prominent among those increased at D10, while mitochondrial and ribosomal proteins are most prominent among those decreased at D10 (Online Resource 4a and 4b).

The majority of proteins identified at D10 were also identified at D5 (28/33 and 29/36) (Fig. 3c, d). Interestingly, among those proteins in the overlapping dataset, 25 of the increased proteins showed higher abundance at D10 compared with D5, while 20 of the decreased proteins showed lower abundance at D10 compared with D5, supporting the notion that these proteins are regulated with age (Fig. 3e, f).

Given that the indirect comparison of protein abundance described above found relatively few proteins to be changed at D10 compared with YA, we complemented these analyses of the aging proteome by also comparing the proteome of YA and D10 animals following heat shock. To identify only those changes that characterize the aging proteome, rather than the response to heat shock, we excluded from this analysis any protein that changed in abundance in response to heat stress at either of the examined life stages (67 proteins in total). This revealed 381 proteins as increased in abundance at D10 compared with YA and 474 proteins as decreased in abundance (Online Resource 3d–3f). Wormbase analysis of the Gene Ontologies of the increased proteins identified nuclear and extracellular as the two most represented cellular component categories, with 48 and 34 proteins, respectively. Among the decreased proteins, mitochondrial and ribosomal were the two most represented categories, with 92 and 73 proteins, respectively (Online Resource 4c and 4d).

Furthermore, these proteomic changes identified in D10 animals overlap substantially with those described above in D5 animals. That is, 160 proteins are increased at both D5 and D10 compared with YA and 247 proteins are decreased at both D5 and D10 compared with YA. Within the set of proteins increased at both D5 and D10 are the replication licensing factors, topoisomerase, nuclear lamin, histone proteins, Transthyretin-Related family proteins and vitellogenins that were described above. Similarly, among the proteins decreased at both D5 and D10 are the myosin-related proteins and proteins involved in mitochondrial proteostasis and ribosomal protein synthesis. In these latter categories, additional proteins were identified as decreased in abundance at D10, including three mitochondrial ribosomal proteins (MRPS-9, MRPS-22, and MRPS-26), translation initiation factors (EIF-1.A, EIF-3.H, IFFB-1, IFG-1, INF-1) and a translation elongation factor (EEF-1G).

### Heat shock proteins and intermediate filaments increase in abundance following heat shock while P granule-associated proteins decrease

**Fig. 4:**
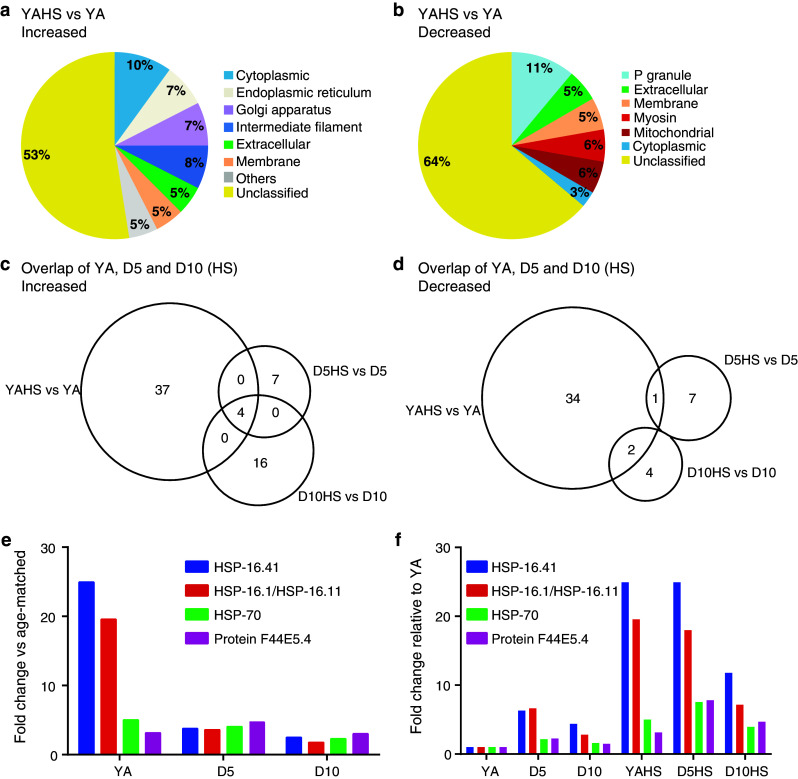
The proteomic profile of *C. elegans* changes with heat shock. Heat shocked samples are indicated by ‘HS’. **a** Gene Ontology (cellular component) profile of proteins increased in abundance in response to heat shock (34 °C/2 h) in young adults (YA). Only the major categories are shown; categories with fewer proteins are cumulatively displayed as ‘Others’. Proteins without a Gene Ontology (cellular component) classification are shown as ‘Unclassified’. **b** Gene Ontology (cellular component) profile of proteins decreased in abundance in response to heat shock in YA. **c** Venn diagram of proteins increased in abundance at YA, D5 and D10 after heat shock compared with their age-matched controls. **d** Venn diagram of proteins decreased in abundance at YA, D5, and D10 after heat shock compared with their age-matched controls. **e** Fold change of selected heat shock proteins increased in abundance at YA, D5 and D10 compared with their age-matched non-heat shocked controls. **f** Fold change of selected heat shock proteins increased in abundance at YA, D5, and D10 (non-heat shocked or HS) compared with YA (non-heat shocked)

We next determined proteomic changes in response to heat shock. At YA stage, 40 proteins were increased and 36 decreased in heat shocked nematodes compared with controls (Online Resource 5a and 5b). Analysis of Gene Ontologies revealed an association of the increased proteins with the following cellular components: cytoplasmic, endoplasmic reticulum, Golgi apparatus, intermediate filament and extracellular (Fig. 4a). The intermediate filament proteins, consisting of IFA-1, MUA-6, and IFC-2, are of particular interest since such proteins are critical in the formation of aggresomes that form in response to protein misfolding [43]. Decreased upon heat shock were proteins associated with the cellular components: P granule, extracellular and mitochondrial (Fig. 4b). The P granule-associated proteins were PGL-1, CGH-1, CAR-1, and GLH-1, and the extracellular proteins included vitellogenins VIT-2 and VIT-6.

While the analysis of cellular component Gene Ontologies had successfully classified the majority of proteins identified as changed in abundance with age, 44 of the 76 proteins changed upon heat shock were not classified using this ontology. Among them were HSPs belonging to several families: hsp70 (HSP-4, HSP-70, F44E5.4), hsp110 (C30C11.4 [44]), hsp90 (DAF-21) and the small HSPs (HSP-16.1, HSP-16.41), which increased in abundance up to 25-fold upon heat shock. Interestingly, several stress-related proteins decreased, including catalases CTL-1 and CTL-2 and small heat shock protein SIP-1.

### The heat shock response diminishes with age

We next compared the proteomic response to heat shock across the three age groups. In this case, since all comparisons were made directly between the proteomes of the heat shocked animals and non-heat shocked counterparts of the same age, and the same statistical thresholds were applied, the scale of the response at each time point can be directly compared. Interestingly, when compared with young adults, fewer proteins changed in abundance in response to heat shock in the aged animals (Fig. 4c, d). Specifically, while at YA 40 proteins were increased and 36 decreased, at D5 only 11 proteins were increased and 8 decreased (Online Resource 5c and 5d). Similar to the profile at D5, at D10, only 16 proteins increased and 6 decreased following heat shock (Online Resource 5e and 5f). Comparing the three time points, we found no proteins that uniformly decreased in abundance after heat shock in both young and old animals. We did, however, identify four HSPs that were increased in both young and old animals: two hsp70s (HSP-70 and F44E5.4) and two small HSPs (HSP-16.1 and HSP-16.41) (Fig. 4c). When their abundance at each time-point following heat shock is expressed relative to the age-matched control, there is a striking reduction in the scale of the increase of these proteins in response to heat shock in D10 animals and this reduction is most marked for the small HSPs (Fig. 4e). We additionally expressed the abundance of these proteins at each time-point relative to the YA control sample and found that all 4 proteins were increased in D5 and D10 control samples (Fig. 4f). However, the diminished scale of induction in response to heat shock means that the abundance of small HSPs following heat shock in D10 animals is reduced to <50 % of the levels attained in young adulthood. Together, these observations suggest that the heat shock response diminishes with age.

### AHA-containing peptides are identified and relative abundance quantified by iTRAQ

We next identified the subset of MS/MS-identified peptides that contain AHA by including ‘AHA modification’ in the ProteinPilot search parameters. This identified 323 AHA-modified peptides corresponding to 205 proteins. It is not surprising that this number is small relative to the number of peptides identified in our global proteomic analysis, since peptides synthesized de novo during the 6-h AHA incubation would represent only a small portion of the total protein pool. Furthermore, the low charging rate and relatively low abundance of methionine mean that not all de novo peptides will be AHA-tagged. The relative abundance of the AHA-tagged peptides was computed as the geometric mean of the iTRAQ ratios from all measurements of a given peptide across the replicate experiments, and <0.83 or >1.2 were fixed as thresholds for decreased and increased abundance, respectively.

### Analysis of AHA-tagged peptides reveals increased vitellogenin synthesis and decreased synthesis of distinct ribosomal, mitochondrial and myosin-related proteins in aged animals

**Fig. 5:**
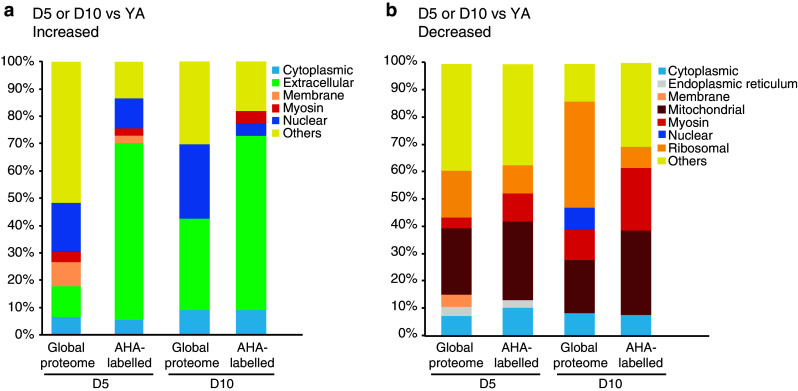
The Gene Ontology profile of newly synthesized proteins in *C. elegans* reflects that of the global proteome. Comparison of Gene Ontology (cellular component) profiles of AHA-labeled peptides and the global proteome for proteins/peptides shown to be **a** increased or **b** decreased in abundance at D5 or D10 of adulthood compared with YA. Percentages shown here are the proportions of proteins/peptides in the global proteome or AHA-labeled data set

We first considered those AHA-tagged peptides that were detected as increased in abundance in D5 aged animals. When we classified these 33 peptides using the cellular component ontology, the most represented components were extracellular and nuclear, mirroring the components that were enriched in our global analysis of proteins with increased abundance at D5 (Fig. 5a). We next examined all AHA-tagged peptides that were detected at D5 and/or D10 (Table 3 and Online Resource 6a) and compared these with the proteins identified in our global analysis. 42 AHA-containing peptides were increased at D5 and/or D10 compared with YA and of these, 31 peptides correspond to proteins that were also increased in abundance in aged animals in our global analysis. These include 23 peptides derived from vitellogenins as well as peptides corresponding to other extracellular proteins such as the transthyretin-like protein TTR-15 and to nuclear proteins such as the histone H4 HIS-1. For these proteins, our analysis of the AHA-tagged protein pool indicates that the observed increased abundance with age is not solely due to accumulation, but rather reflects a relative increase in synthesis.

**Table 3:** AHA-labeled peptides increased in abundance in day 5 and/or day 10 animals compared with young adults

UniProt	Gene		Name	D5 vs. YA			D10 vs. YA		
				*N*	Fold change (range)		*N*	Fold change (range)	
					Max	Min		Max	Min
Extracellular									
Vitellogenins									
P55155; P05690	vit-1; vit-2		Vitellogenin-1, -2	20	13.7	10.0	13	13.5	6.7
P55155; P05690	vit-1; vit-2		Vitellogenin-1, -2	7	2.1	1.4			
P05690	vit-2		Vitellogenin-2	132	11.6	7.0	143	12.2	4.0
Q9N4J2; P18947; P06125	vit-3; vit-4; vit-5		Vitellogenin-3, -4, -5	20	20.2	17.0	13	18.1	10.5
Q9N4J2; P18947; P06125	vit-3; vit-4; vit-5		Vitellogenin-3, -4, -5	10	11.1	8.4	7	17.8	6.9
P18947; P06125	vit-4; vit-5		Vitellogenin-4, -5	1	8.6	5.5			
P06125	vit-5		Vitellogenin-5	2	10.8	6.5	1	9.6	3.4
P06125	vit-5		Vitellogenin-5	1	6.4	4.5	2	6.3	2.8
P18948	vit-6		Vitellogenin-6	21	18.6	12.1	82	12.5	3.6
P18948	vit-6		Vitellogenin-6				17	12.5	3.0
Transthyretins									
Q22288	ttr-15		Transthyretin-related family domain	3	2.0	1.4	5	2.2	1.3
Chitin metabolic processes									
P41996	cpg-2		Chondroitin proteoglycan-2	1	2.1	1.2			
Nuclear									
Histones									
P62784	his-1		Histone H4	2	5.6	4.8			
Others									
P42168	kin-19		Casein kinase I isoform alpha	1	3.9	2.1			
P49029	mag-1		*Drosophila* mago nashi homolog	7	1.7	1.3			
Q18240	spr-2		Suppressor of presenilin-2	1	2.2	1.6			
Other cellular components									
O01541	aars-2		Alanyl amino-acyl tRNA synthetase				1	1.7	1.2
Q18493	C36A4.4		Orthologous to human isoform AGX2	4	2.0	1.5	1	2.3	1.5
Q18688	daf-21		Abnormal dauer formation, heat shock protein 90	3	2.4	1.5			
O44400	F37C4.5		Protein F37C4.5	1	1.9	1.6			
O17271	heh-1		HE1 homologue				1	3.1	1.6
P90900	ifa-4		Intermediate filament, A	1	2.8	1.8	2	4.6	2.0
Q965W1	lbp-9		Lipid binding protein	9	1.8	1.5	6	2.2	1.5
Q93572	rla-0		60S acidic ribosomal protein P0	2	2.0	1.4			
Q19200	sto-1		Stomatin-1	1	5.9	3.9			
P52275	tbb-2		Tubulin beta-2 chain				1	3.7	1.6
Q27535	ZC434.8		Orthologous to human creatine kinase B-type	1	1.6	1.5			
Q23629	ZK836.2		Orthologous to human 2-oxoglutarate dehydrogenase E1	1	2.6	1.8			

Multiple entries of the same accession number refer to unique peptides identified by mass spectrometry for the same protein. For each identified protein, only the two most frequently detected peptides are shown here

*N* represents the number of times each peptide was identified

We next considered those AHA-tagged peptides that were detected as decreased in abundance in D5 aged animals. When we classified these 38 peptides using the cellular component ontology, the most represented components were mitochondrial, myosin-related, ribosomal and cytoplasmic, mirroring the components that were enriched in our global analysis of proteins showing decreased abundance at D5 (Fig. 5b). We then examined all AHA-tagged peptides that were detected as decreased at D5 and/or D10 (Table 4 and Online Resource 6b) and compared these with the proteins identified in our global analysis. Fifty-three AHA-containing peptides were decreased at D5 and/or D10 compared with YA and of these, 42 peptides are derived from proteins that were also decreased in abundance in aged animals in our global analysis. These include 6 from ribosomal (including RPS-17, RPL-23, UBL-1), 15 from mitochondrial (including SDHA-1, MDH-1, FUM-1, and ATP-2) and 8 from myosin-related proteins (MYO-1, MYO-2, MLC-1/2/3, UNC-15, UNC-54). For these proteins, their relative decrease in abundance with age is not solely due to degradation but additionally reflects a relative decrease in their synthesis.

**Table 4:** AHA-labeled peptides decreased in abundance in day 5 and or day 10 animals compared with young adults

UniProt	Gene	Name		D5 vs. YA			D10 vs. YA		
				*N*	Fold change (range)		*N*	Fold change (range)	
					Max	Min		Max	Min
Mitochondrial									
Electron transport chain									
P46561	atp-2	ATP synthase subunit beta		5	0.8	0.6			
P46561	atp-2	ATP synthase subunit beta		4	0.7	0.4			
Q9XXK1	H28O16.1	ATP synthase subunit alpha, mitochondrial		1	0.5	0.3	3	0.8	0.5
Q9XXK1	H28O16.1	ATP synthase subunit alpha, mitochondrial					8	0.6	0.4
Chaperones									
P50140	hsp-60	Chaperonin homolog, Hsp60 family		2	0.6	0.4			
Tricarboxylic acid (TCA) cycle-related									
O17214	fum-1	Fumarate hydratase		16	0.8	0.6			
O02640	mdh-1	Malate dehydrogenase					8	0.8	0.6
Q09508	sdha-1	Succinate dehydrogenase complex subunit A		1	0.7	0.4			
Q09508	sdha-1	Succinate dehydrogenase complex subunit A		1	0.8	0.6			
Others									
P52713	alh-8	Aldehyde dehydrogenase		5	0.8	0.6	1	0.6	0.4
O45228	B0513.5	Orthologous to human isoform 1 of proline dehydrogenase 1		2	0.7	0.4			
Q18040	C16A3.10	Orthologous to human ornithine aminotransferase,					2	0.8	0.4
Q22111	mmaa-1	Methylmalonic aciduria type A protein		4	0.7	0.4	4	0.8	0.3
Q19842	pcca-1	Propionyl coenzyme-A carboxylase alpha subunit		4	0.8	0.6			
Q9U2M4	Y38F1A.6	Ortholog, human isoform 1, HOT					1	0.6	0.3
Q9U2M4	Y38F1A.6	Ortholog, human isoform 1, HOT					5	0.8	0.3
Ribosomal									
Q9N4I4	rpl-1	60S ribosomal protein L10a		1	0.7	0.5			
P48158	rpl-23	60S ribosomal protein L23					7	0.8	0.4
O01692	rps-17	40S ribosomal protein S17		5	0.8	0.6			
P37165	ubl-1	Ubiquitin-like protein 1, 40S ribosomal protein S27a		2	0.7	0.6			
O01504	C37A2.7	60S acidic ribosomal protein P2					6	0.8	0.5
Q93572	rpa-0	60S acidic ribosomal protein P0		1	0.8	0.4			
Myosins									
P19626; P19625	mlc-2; mlc-1	Myosin regulatory light chain-1. -2		4	0.5	0.3	2	0.5	0.3
P53014	mlc-3	Myosin, essential light chain		4	0.8	0.6			
P02567	myo-1	Myosin-1		1	0.7	0.5	1	0.7	0.3
P12845	myo-2	Myosin-2					8	0.7	0.4
P10567	unc-15	Paramyosin					14	0.7	0.3
P10567	unc-15	Paramyosin					3	0.4	0.2
P02566	unc-54	Myosin-4					8	0.7	0.4
Other cellular components									
Q23500	aco-1		Aconitase	3	0.7	0.5			
P27604	ahcy-1		*S*-adenosylhomocysteine hydrolase homolog	22	0.6	0.4	6	0.5	0.3
P27604	ahcy-1		*S*-adenosylhomocysteine hydrolase homolog	20	0.7	0.5	8	0.6	0.4
P54216	aldo-1		Fructose bisphosphate aldolase	6	0.7	0.4			
P54216	aldo-1		Fructose bisphosphate aldolase	10	0.8	0.5			
Q09936	C53C9.2		Orthologous to human myelin transcription factor 1	1	0.6	0.3			
P27798	crt-1		Calreticulin				71	0.7	0.4
Q10576	dpy-18		Prolyl 4-hydroxylase subunit alpha-1	9	0.8	0.5	1	0.6	0.2
Q18164	dpyd-1		Dihydropyrimidine dehydrogenase				5	0.6	0.2
P53013	eef-1A.1		Elongation factor 1-alpha	87	0.6	0.5			
Q10454	F46H5.3		Orthologous to human creatine kinase M-type	3	0.7	0.4			
P34689	glh-1		Germ line helicase	1	0.5	0.3			
P04970	gpd-1		Glyceraldehyde 3-phosphate dehydrogenase 1	59	0.6	0.5			
P17329	gpd-2		Glyceraldehyde 3-phosphate dehydrogenase 2	16	0.6	0.4			
P17330	gpd-3		Glyceraldehyde 3-phosphate dehydrogenase	9	0.7	0.6	6	0.6	0.5
P17331	gpd-4		Glyceraldehyde 3-phosphate dehydrogenase 4	22	0.6	0.5			
O18054	pfd-3		Prefoldin subunit 3	1	0.7	0.5			
Q9TZQ3	pgl-1		P-granule abnormality	2	0.5	0.3			
P34453	prx-19		Peroxisome biogenesis factor 19				1	0.7	0.4
Q21215	rack-1		RACK1 (mammalian receptor of activated C kinase) homolog	2	0.6	0.3			
Q17334	sodh-1		Sorbitol dehydrogenase family				2	0.7	0.4

Multiple entries of the same accession number refer to unique peptides identified by mass spectrometry for the same protein. For each identified protein, only the two most frequently detected peptides are shown here

*N* represents the number of times each peptide was identified

*HOT* hydroxy acid-oxoacid transhydrogenase

### Analysis of AHA-tagged peptides affirms a decrease in the heat shock response with age

**Fig. 6:**
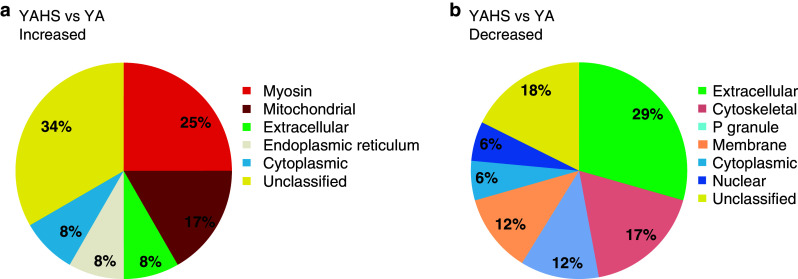
The proteomic profile of newly synthesized proteins in *C. elegans* changes in response to heat shock. Gene Ontology (cellular component) profile of AHA-modified peptides shown to be **a** increased or **b** decreased in abundance in response to heat shock (34 °C/2 h) in YA. Proteins without a Gene Ontology (cellular component) classification are shown as ‘Unclassified’

To complement our global analysis of the heat shock response, we secondly examined changes to AHA-labeled peptides in response to heat shock and identified 12 increased and 17 decreased peptides in YA animals (Fig. 6a, b and Online Resource 7a and 7b). Increased were AHA-labeled peptides corresponding to the categories mitochondrial (ZK836.2 and GAS-1) and myosin-related (UNC-54 and UNC-15), and HSPs [HSP-3 (hsp70) and C30C11.4 (hsp110)]. The nascent polypeptide-associated complex (NAC) alpha subunit, ICD-2 was also increased in response to heat shock. This is particularly interesting because this complex is a key regulator of proteostasis [31]. Not detected were other HSPs that were dramatically increased in response to heat shock through our global proteome analysis, possibly because of the timing of the addition of AHA and a lag before incorporation. Among the AHA-labeled proteins decreased after heat shock were vitellogenins (VIT-2, -5, -6), P granule components (PGL-1 and GLH-1) and cytoskeletal proteins (ACT-1/2/4, TBA-2 and TBB-2).

Examining the AHA-labeled peptides that changed in abundance in D5 and D10 heat shocked nematodes revealed fewer labeled peptides in aged animals, 25 at D5 and 14 at D10, compared with 29 at YA (Online Resource 7c-f). Interestingly, no AHA-labeled peptides corresponding to heat shock proteins were detected as increased in abundance relative to control at either D5 or D10. As indicated by our global proteomic analysis, these analyses of de novo synthesized proteins suggest that the response to heat shock is diminished in aged animals.

## Discussion

To obtain a snapshot of the aging process and the stress response at the level of the proteome, we subjected young and aged *C. elegans* to quantitative proteomics and determined both changes in total protein abundance as well as specific changes in the pool of de novo synthesized proteins using ‘click chemistry’. These profiles collectively suggest specific changes in the protein control system as the worms age.

### The proteome of aged animals contains abundant yolk proteins and suggests reduced mitochondrial function and sarcopenia

In addition to the ribosomal proteins that we discuss separately below, we observed major alterations in the abundance and synthesis of three key groups of proteins that represent an altered proteomic profile with aging: vitellogenins, mitochondrial and myosin-related proteins. The vitellogenins are yolk proteins that, synthesized in the nematode intestine, are secreted into the body cavity and taken up by the gonad to provision developing oocytes [45]. We observed a significant increase in both the abundance and de novo synthesis of vitellogenins in aged animals, supporting the finding that yolk accumulation in the body cavity is a marker of aging [46]. Yolk proteins fall into three groups: YP170, formed from proteins encoded by *vit*-*1,* -*2,* -*3,* -*4,* and -*5*; YP115 and YP88, both derived by cleavage from a *vit*-*6*-encoded precursor [47]. VIT-6 was previously identified as a major carbonylated protein in aged worms, suggesting decreased turnover due to oxidative damage [48]. Evidence for a role of vitellogenins in the aging process comes from several observations: down-regulating *vit*-*2* and *vit*-*5* increases lifespan [49], long-lived *daf*-*2* mutants have decreased yolk proteins [50], and knockdown of several factors that increase lifespan also decreases vitellogenins [51]. Why accumulation of yolk facilitates aging is not clear, although lipotoxicity due to ectopic deposition may have a role [52].

We found aged animals to be additionally characterized by reductions in mitochondrial proteins, both globally and considering the de novo synthesized pool. These mitochondrial proteins included numerous components of the ETC and enzymes of the TCA cycle. Changes to mitochondrial structure and function during aging have previously been reported in *C. elegans*, including enlargement of mitochondria and decreased activity of the ETC in aged animals [53]. Altered mitochondrial metabolism also characterizes age-associated neurodegeneration [30, 54]. Therefore, a reduction in mitochondrial activity, such as in supplying cellular energy, may characterize both pathological and physiological aging.

Aged animals were also characterized by reduced abundance and reduced synthesis of several myosin-related proteins. An age-associated decline in muscle integrity, sarcopenia, has been observed in *C. elegans* [46] and a reduction in the levels of myosin-related proteins is consistent with this. Interestingly, a recent proteomic analysis revealed an increase in the abundance of several muscle proteins in long-lived worms compared with chronologically aged matched wild-type counterparts [55]. In addition to components of the contractile sarcomere that we have identified as reduced in aged animals, increases in other proteins relevant to muscle activity were noted in the long-lived worms, including mitochondrial creatine kinase (W10C8.5) and calsequestrin (CSQ-1) [55], both of which were also reduced in our analysis of aged animals.

### Reduced ribosomal proteins, reduced mitochondrial chaperones, and diminished heat stress response in aging suggest perturbed proteostasis in old animals

It was observed several decades ago in the free-living nematode *Turbatrix* *aceti*, by measuring the incorporation of radiolabeled leucine, that protein synthesis was slowed in old animals [32]. More recently, analysis of polysomes revealed that global rates of protein synthesis decline during aging in *C. elegans* [31]. Consistent with this, we observed an age-dependent decrease in ribosomal subunits using quantitative proteomics; both the total abundance of numerous ribosomal proteins, as well as their representation in the de novo pool, was found to decrease with age. While this suggests decreased synthesis with age, other factors that contribute to the observed decreases could be increased degradation and/or reduced solubility. Since our extraction protocol was not designed to investigate the insoluble proteome, those proteins that shift to this pool with age would be identified as decreased in our analysis of soluble proteins. Indeed of the proteins for which we identified reduced levels of AHA-labeled peptides in aged animals, one-third show increased insolubility with age as published previously [12]. In either case, together with aggregation-related toxicity, the removal of these proteins from the functional protein pool is likely to contribute to the aging process.

The contribution of globally reduced translation to proteostasis in aging is intriguing. Reduced protein synthesis may contribute to proteostasis by reducing levels of damaged proteins and hence reducing the chaperone load [31]. Another possibility is that the energy savings associated with global reduction in translation enable cells to redirect resources towards maintenance and repair [9]. Reduction of global protein synthesis is likely to be important in aging since reduced translation increases lifespan [8–10]. Lifespan extension is also achieved by knocking down a single translation initiation factor that results in differential translation by changing ribosomal loading [56]. A recent comparison of the proteome of wild-type animals with that of long-lived *daf*-*2* mutants, which carry a mutation in the Insulin/Insulin-like Growth Factor (IGF-1) receptor, revealed a role for protein translation in Insulin/IGF-1-mediated lifespan regulation [57]. Interestingly, this study found marked reductions both in the abundance of numerous ribosomal proteins and in polysomal protein translation in *daf*-*2* mutants at day 1 of adulthood compared with wild-type animals of the same age [57]. Similarly, in a separate study, global rates of protein synthesis were recently shown to be reduced in both *daf*-*2* mutants and in diet-restricted worms [55]. Our finding of reduced abundance of ribosomal proteins in aged wild-type animals appears then to represent a beneficial change that occurs in the aging proteome, contrasting, for example, with increased levels of vitellogenins, which represents an apparently detrimental change.

Beyond alterations to the translational machinery, our proteomic data also indicate changes in mitochondrial proteostasis with age. Decreased abundance and decreased synthesis of several mitochondrial chaperones, including HSP-6 and HSP-60, was detected in aged animals. Decreased abundance of both HSP-6 and HSP-60 in 20 day old wild-type animals has previously been reported [58] and a role for *hsp*-*6* in longevity regulation is suggested by the findings that knockdown of *hsp*-*6* reduces lifespan [58] and that numerous treatments that extend lifespan induce the expression of *hsp*-*6* [22, 59]. Our analyses reveal that additional components of the mitochondrial proteostasis network are also decreased during aging, including ATAD-3 and the prohibitins PHB-1 and PHB-2. Mammalian ATAD3 and prohibitins interact with mitochondrial nucleoids, structures containing condensed mitochondrial DNA, and are, among several functions, required for mitochondrial protein synthesis [60]. The prohibitins have previously been identified as longevity determinants in *C. elegans*; while knockdown of PHB-1 or PHB-2 reduces the lifespan of wild-type *C. elegans*, the lifespan of a range of *C. elegans* mutants, including the long-lived *daf*-*2* mutant, is increased by prohibitin depletion [61]. Knockdown of ATAD-3 increases the lifespan of wild-type *C.* *elegans* [62]. These observations highlight the importance of the mitochondrial proteostasis network during aging.

Our finding of reduced ribosomal proteins and mitochondrial chaperones in aged animals suggests that proteostasis is altered during aging, and our observation of a decrease in heat stress-responsiveness in aged animals provides further evidence for this. As in earlier studies examining the induction of gene transcription [63, 64] and protein expression [65, 66] in the nematode in response to heat shock, our proteomic analysis identified several HSPs as being significantly induced in response to heat shock in YA animals. We also observed that basal levels of expression of certain HSPs increased in aged animals, as has previously been reported in both *C. elegans* [67] and *Drosophila* [68]. Despite this basal increase, we observed a dramatic reduction in the induction of these HSPs in response to heat shock in aged animals. This mirrors measures of the transcriptional response to heat shock, which found that the induction of genes encoding several hsp70s and small HSPs was diminished by day 4 of adulthood [69], and the observation that HSP-16 induction following heat shock is diminished in aged animals compared with young adults [67, 70]. Furthermore, thermotolerance, as measured by nematode survival at 35 °C, is reduced in aged animals [69, 71]. Together, these findings indicate that the capacity of animals to respond to proteotoxic stress diminishes with age.

### Future directions and conclusion

In the current study, we determined both the global and de novo proteome using quantitative mass spectrometry. By coupling the proteins that have incorporated AHA with a biotin-FLAG-alkyne tag, followed by enrichment via the biotin moiety, in future studies, the AHA-modified pool could be significantly increased by enriching specifically for these peptides [25]. Furthermore, given the increased propensity of proteins to aggregate with aging, extending the proteomic analysis to the insoluble protein fraction would provide an additional layer of information.

Tissues vary in their capacity to respond to protein damage during aging, with neurons appearing to be particularly sensitive [72]. Cell type-specific differences in chaperone activity may contribute to this variation [73]. Targeted incorporation of the bio-orthogonal label and identification by ‘click chemistry’ in defined cell types and tissues could be achieved by selective expression of a modified tRNA synthetase [74]. Such a focused analysis could provide further information on the role de novo protein synthesis has in the response to age-associated cellular damage in specific tissues.

Together, our work provides a proteomic snapshot of aging and evidence that the proteostasis network is altered in aged animals, both in physiological conditions and in response to heat stress. In addition, we have established for the first time a ‘click chemistry’ protocol in *C. elegans* that offers the possibility, with the same tagging method, to firstly visualize in which cells and subcellular compartments de novo protein synthesis has occurred and secondly to identify the newly synthesized proteins. Since nematodes are amenable to experimentation using a variety of physical and chemical stressors, our protocol enables a powerful whole organismal analysis of proteomic changes in response to these stresses.

### Electronic supplementary material

Below is the link to the electronic supplementary material.

Treatment with AHA does not result in toxicity as measured by (a) the thrashing assay or (b) induction of the mitochondrial UPR. For the thrashing assay, wild-type (N2) worms were used. To assay for induction of the mitochondrial UPR, fluorescence intensity was measured using a Phsp-6::gfp(zcIs13) reporter line. Incubations with 0 or 2 mM AHA were carried out for both experiments in liquid culture for 6 hours at room temperature. For (b), a 50 mM paraquat treatment under the same conditions was used as a positive control for induction of GFP. Both experiments were conducted in duplicate (n=20 per duplicate). Error bars indicate mean +/- SEM. Statistical analysis was conducted using the one-way ANOVA test (Dunnet’s post-test). pvalues are indicated by ns = not significant, *<0.05 (EPS 1600 kb)

2a: Spreadsheet of proteins showing increased abundance in day 5 animals when compared with young adults 2b: Spreadsheet of proteins showing decreased abundance in day 5 animals when compared with young adults 2c.1: Breakdown by worm Gene Ontology (cellular component) of proteins increased at D5 when compared with YA. 2c.2: Breakdown by human Gene Ontology (cellular component) of unclassified proteins increased at D5 when compared with YA. 2c.3: Breakdown by worm Gene Ontology (cellular component) of proteins decreased at D5 when compared with YA. 2c.4: Breakdown by human Gene Ontology (cellular component) of unclassified proteins increased at D5 when compared with YA (XLSX 76 kb)

3a: Spreadsheet of proteins showing increased abundance in day 10 animals when compared with young adults 3b: Spreadsheet of proteins showing decreased abundance in day 10 animals when compared with young adults 3c.1: Breakdown by worm Gene Ontology (cellular component) of proteins increased in D10 animals when compared with YA. 3c.2: Breakdown by human Gene Ontology (cellular component) of unclassified proteins increased in D10 animals when compared with YA. 3c.3: Breakdown by worm Gene Ontology (cellular component) of proteins decreased in D10 animals when compared with YA. 3c.4: Breakdown by human Gene Ontology (cellular component) of unclassified proteins decreased in D10 animals when ompared with YA. 3d: Spreadsheet of proteins showing increased abundance in D10 animals when compared with young adults; proteins increased in D10 heat shocked animals were compared with young adult heat shocked animals, excluding proteins showing changed abundance due to heat shock. 3e: Spreadsheet of proteins showing decreased abundance in D10 animals when compared with young adults; proteins decreased in D10 heat shocked animals were compared with young adult heat shocked animals, excluding proteins showing changed abundance due to heat shock. 3f.1: Breakdown by worm Gene Ontology (cellular component) of proteins increased in D10 heat shocked animals were compared with YA heat shocked animals, excluding proteins showing changed abundance due to heat shock. 3f.2: Breakdown by human Gene Ontology (cellular component) of unclassified proteins increased in D10 animals when compared with YA. 3f.3: Breakdown by worm Gene Ontology (cellular component) of proteins decreased in D10 heat shocked animals were compared with YA heat shocked animals, excluding proteins showing changed abundance due to heat shock. 3f.4: Breakdown by human Gene Ontology (cellular component) of unclassified proteins decreased in D10 animals when compared with YA (XLSX 118 kb)

4a: Gene Ontology (cellular component) profile of proteins showing increased abundance in D10 animals when compared with young adults. Only the major categories are shown; categories with fewer proteins are cumulatively displayed as ‘Others’. Proteins without a Gene Ontology (cellular component) classification are shown as ‘Unclassified’. 4b: Gene Ontology (cellular component) profile of proteins showing decreased abundance in D10 animals when compared with young adults. 4c: Gene Ontology (cellular component) profile of proteins showing increased abundance in heat shocked D10 animals when compared with heat shocked young adults, excluding proteins showing changed abundance due to heat shock. 4d: Gene Ontology (cellular component) profile of proteins showing decreased abundance in heat shocked D10 animals when compared with heat shocked young adults, excluding proteins showing changed abundance due to heat shock (XLSX 19 kb)

5a: Spreadsheet of proteins showing increased abundance in heat shocked young adult animals when compared with age-matched controls 5b: Spreadsheet of proteins showing decreased abundance in heat shocked young adult animals when compared with age-matched controls 5c: Spreadsheet of proteins showing increased abundance in heat shocked D5 animals when compared with age-matched controls 5d: Spreadsheet of proteins showing decreased abundance in heat shocked D5 animals when compared with age-matched controls 5e: Spreadsheet of proteins showing increased abundance in heat shocked D10 animals when compared with age-matched controls 5f: Spreadsheet of proteins showing decreased abundance in heat shocked D10 animals when compared with age-matched controls (XLSX 33 kb)

6a: Spreadsheet of AHA-modified peptides showing increased abundance in D5 and/or D10 animals when compared with young adults 6b: Spreadsheet of AHA-modified peptides showing decreased abundance in D5 and/or D10 animals when compared with young adults (XLSX 29 kb)

7a: Spreadsheet of AHA-modified peptides showing increased abundance in heat shocked young adults when compared with age-matched controls 7b: Spreadsheet of AHA-modified peptides showing decreased abundance in heat shocked young adults when compared with age-matched controls 7c: Spreadsheet of AHA-modified peptides showing increased abundance in heat shocked D5 animals when compared with age-matched controls 7d: Spreadsheet of AHA-modified peptides showing decreased abundance in heat shocked D5 animals when compared with age-matched controls 7e: Spreadsheet of AHA-modified peptides showing increased abundance in heat shocked D10 animals when compared with age-matched controls 7f: Spreadsheet of AHA-modified peptides showing decreased abundance in heat shocked D10 animals when compared with age-matched controls (XLSX 30 kb)

Supplementary methods (DOCX 23 kb)
