# Supplementary material for: Altered proteostasis in aging and heat shock response in C. elegans revealed by analysis of the global and de novo synthesized proteome
Source: Cell Mol Life Sci. 2014 Jan 24;71(17):3339–61. doi: 10.1007/s00018-014-1558-7 (PMC4131143; doi:10.1007/s00018-014-1558-7)
Supplement: Supplementary file 8 — Supplementary methods (DOCX 23 kb) [file 18_2014_1558_MOESM8_ESM.docx]

**Supplementary methods**

**iTRAQ quantitative mass spectroscopy and data analysis**

Nematodes were resuspended in protein extraction buffer (0.25 M TEAB (triethylammonium bicarbonate buffer, T7408, Sigma), 2% SDS (v/v) buffer) in the presence of a protease inhibitor cocktail (Sigma), sonicated for two cycles of 20 and 30 sec, respectively, then placed on ice. This was followed by centrifugation at 12,000 *g* for 5 min at 4°C. The supernatant was collected and 75 µg protein from each sample was reduced at 60 °C with a final concentration of 5 mM TCEP (Tris(2-carboxyethyl)phosphine hydrochloride) for 1 h, and alkylated with a final concentration of 10 mM MMTS (S-Methyl methanethiosulfonate) for 10 min at RT. The sample was diluted with 0.25 M TEAB to adjust the SDS concentration to 0.05% prior to digestion with 3 µg trypsin for 16 h at 37°C. The sample was then lyophilized in a centrifugal vacuum dryer and dissolved in 30 µL 500 mM TEAB.

70 µL of ethanol was added to each of the four iTRAQ 4-plex peptide labelling reagents (114, 115, 116 and 117; AB Sciex) and then one of these reagents each added to the respective sample, followed by incubation at RT for 1 h. After quenching the reaction with 70 µL milliQ water, the four separately labelled samples were pooled and lyophilized. The sample was resuspended in 7 mL SCX buffer A (5 mM KH_2_PO_4_ (Sigma), 25% acetonitrile (Merck), pH 2.7) and loaded onto an SCX column (Agilent 1100 quaternary HPLC system with Polysulfoethyl A, 200 mm × 2.1 mm, 5 µm, 200Å column). After washing with buffer A, buffer B (5 mM KH_2_PO_4_, 350 mM KCl (Sigma), 25% acetonitrile, pH 2.7) was increased from 10% to 45% over the course of 70 min and then quickly to 100%, where it was maintained for 10 min at a flow rate of 300 µL/min. The SCX fractions were collected every 2 min at the beginning of the gradient for 11 fractions, then at 4 min intervals for the remaining fractions.

After pre-fractionation, the SCX fractions were analysed by nanoLC-ESI-MS/MS. Specifically, the SCX fractions were resuspended in 100 µL loading buffer (0.1% formic acid (FA, Fluka) and 2% acetonitrile) and injected as 40 µL samples into a C18 peptide trapping column (Michrome peptide Captrap) for pre-concentration, followed by desalting with loading buffer at 5 µL/min for 10 min. The peptide trap was then switched into line with the analytical column (SGE ProteCol C18, 300Å, 3μm, 150μm × 10 cm). The peptides were eluted from the column using a linear solvent gradient, from buffer A (0.1% FA): buffer B (0.1% FA and 90% acetonitrile) = 98:2 to buffer A: buffer B = 65:35 at a flow rate of 600 nL/min over a 100 min period. After peptide elution, the column was cleaned with 95% buffer B for 15 minutes and then equilibrated with buffer A for 25 min before the next sample was injected.

A TOFMS (time-of-flight mass spectrometer) survey scan was acquired from 400 to 1,500 *m/z* for 0.25 sec, with the ten most intense multiply charged ions (counts >150) in the survey scan sequentially subjected to MS/MS analysis. Each MS/MS spectrum was acquired for 200 msec in the mass range from 100 to 1500 *m/z* with a total cycle time of approximately 2.3 sec.

The nanoLC ESI MS/MS data were submitted to ProteinPilot V4.2 (AB Sciex) using the combined Fasta database of ‘C. elegans’ and ‘E. coli’ downloaded from PubMed (UniProt database). In the Specify Processing tab, iTRAQ 4-plex peptide labelled for Sample Type, MMTS for Cysteine Alkylation, Quantitation, Bias Correction, Thorough ID and Run False Discovery Rate Analysis were ticked. ‘Protein unused score’ better than 1.3 (better than 95% confidence) was used as the threshold for protein identification. ‘Reverse database decoy analysis’ was used to estimate the protein identification false discovery rate. Using the specified search parameters, iTRAQ ratios and corresponding P-values were only reported for proteins identified by more than two peptides with iTRAQ reporting ion intensity signal to noise ratio > 10. The iTRAQ ratio was calculated as the geometric mean of peptide iTRAQ ratios and represents protein relative abundance. Thresholds for decreased and increased abundance were set at <0.83 and >1.2, respectively. The P-value was calculated using Stouffer’s z-test, which enables P-values from several protein ratios belonging to the same analysis group to be combined, generating a single measure of significance which takes into account the individual peptide ratios used to generate the respective protein ratio ([Whitlock 2005](#_ENREF_45)).   P<0.05 was considered significant. This significance measure was coupled with two additional requirements for a protein to be reported as changed in abundance. First, when a protein ratio indicated an increase in abundance, each of the peptide ratios contributing to that protein ratio must also indicate an increase in abundance, and vice versa. Second, when the geometric mean of protein iTRAQ ratios indicated an increase in abundance, each of the protein iTRAQ ratios from replicate measurements must also indicate an increase in abundance, and vice versa.

The relative abundance of proteins at D10 vs YA were calculated indirectly since these two conditions were not assayed in the same iTRAQ 4-plex experiment. The D10 vs YAHS and YA vs YAHS ratios were calculated directly as described above and then the former divided by the latter to yield the D10 vs YA ratio. In this case a P-value was calculated using a Student’s t-test and P<0.05 was considered significant.

AHA-containing peptides were identified by including “AHA modification” in the ProteinPilot search parameters, with the modification probability set to 90%. For each measurement of a given peptide, ProteinPilot reports an iTRAQ ratio and percentage error. To compute the relative abundance of peptides, the geometric mean of the iTRAQ ratios from all measurements of a given peptide across the replicate experiments was calculated. The geometric means of the upper and lower bounds of the peptide iTRAQ ratios from all measurements of a given peptide were also calculated with reference to the reported percentage error. Peptides for which the mean lower bound of the iTRAQ ratio was >1.2 were considered increased in abundance, while peptides for which the mean upper bound of the iTRAQ ratio was <0.83 were considered decreased in abundance.

**iTRAQ experimental set-up.** The sample identified as ‘Mix YA’ is a pool of three replicate YA heat shocked samples and was used as an internal reference.

| Experiment # | Stage | Heat shock | iTRAQ label |
| --- | --- | --- | --- |
| 1 | Mix YA | + | 114 |
|  | YA | - | 115 |
|  | D5 | + | 116 |
|  | D5 | - | 117 |
| 2 | Mix YA | + | 114 |
|  | D10 | + | 115 |
|  | D10 | - | 116 |
|  | YA | + | 117 |
| 3 | Mix YA | + | 114 |
|  | YA | - | 115 |
|  | D5 | + | 116 |
|  | D5 | - | 117 |
| 4 | Mix YA | + | 114 |
|  | D10 | + | 115 |
|  | D10 | + | 116 |
|  | D10 | - | 117 |

**Quantification of P*hsp-6:gfp* fluorescence**

SJ4100 *zcIs13[hsp-6::GFP]* animals were synchronised by hypochlorite treatment and cultured for two generations in liquid S-medium at room temperature. Animals were fed HB101 bacteria during culture. At young adult stage, worms were incubated with AHA for six hours at the desired concentrations, in duplicate. Prior to imaging, animals were mounted in 0.1% tetramisole (Sigma) in M9 buffer onto agarose pads. 20 images were captured per duplicate. All measurements were conducted in duplicate except for the 50 mM paraquat control. Fluorescence was quantified using ImageJ software.

**Thrashing assay**

Wild-type (N2) animals were synchronised by hypochlorite treatment and cultured for two generations in liquid S-medium at room temperature. Animals were fed HB101 bacteria. At young adult stage, worms were incubated with AHA for six hours at the desired concentrations, in duplicate. Animals were then picked individually into a drop of M9+0.1% Triton-X100 and movements recorded for one minute. 20 worms were analysed per treatment, per duplicate. Videos were captured using a Canon S80 camera at 15frames per second and the number of body bends per minute analysed using the “wrMTrck” plug-in (JS Pedersen) for ImageJ.

Altered proteostasis in aging and heat shock response in *C. elegans* revealed by analysis of the global and *de novo* synthesised proteome

Cellular and Molecular Life Sciences

Vanessa Liang^1†^, Milena Ullrich^1,2†^, Hong Lam^3†^, Yee Lian Chew^3^, Samuel Banister^4^, Xiaomin Song^5^, Thiri Zaw^5^, Michael Kassiou^4^, Jürgen Götz^1,6‡^, Hannah R. Nicholas^3‡^

^1^Brain and Mind Research Institute, University of Sydney, Camperdown, Australia; ^2^Institute for Integrative Neuroanatomy, Charité, Universitätsmedizin Berlin, Berlin, Germany; ^3^School of Molecular Bioscience, University of Sydney, Camperdown, Australia; ^4^Drug Discovery Research Laboratory, University of Sydney, Camperdown, Australia; ^5^Australian Proteome Analysis Facility, Macquarie University, Sydney, Australia; ^6^Centre for Ageing Dementia Research (CADR), Queensland Brain Institute (QBI), The University of Queensland, Brisbane, Australia

Correspondence: Hannah Nicholas

Email: hannah.nicholas@sydney.edu.au
